# Design and methods of the Healthy Kids & Families study: a parent-focused community health worker-delivered childhood obesity prevention intervention

**DOI:** 10.1186/s40608-019-0240-x

**Published:** 2019-06-03

**Authors:** Amy Borg, Christina F. Haughton, Mullen Sawyer, Stephenie C. Lemon, Kevin Kane, Lori Pbert, Wenjun Li, Milagros C. Rosal

**Affiliations:** 1 Division of Preventive and Behavioral Medicine, University of Massachusetts Medical School, 55 Lake Avenue North, Worcester, MA 01655 USA; 2 Oak Hill Community Development Corporation, 74 Providence Street, Worcester, MA 01604 USA; 3 Tufts University Friedman School of Nutrition and Policy, 150 Harrison Avenue, Boston, MA 02111 USA

**Keywords:** Childhood obesity prevention, Community health worker, Parent-focused intervention

## Abstract

**Background:**

One third of U.S. children and two thirds of adults are overweight or obese. Interventions to prevent obesity and thus avert threats to public health are needed. This paper describes the design and methods of the Healthy Kids & Families study, which tested the effect of a parent-focused community health worker (CHW)-delivered lifestyle intervention to prevent childhood obesity.

**Methods:**

Participants were English or Spanish-speaking parent-child dyads (*n* = 247) from nine elementary schools (grades K-6) located in racial/ethnically diverse low-income communities in Worcester, Massachusetts. Using a quasi-experimental design with the school as the level of allocation, the study compared the lifestyle intervention vs. an attention-control comparison condition. The lifestyle intervention was guided by social cognitive theory and social ecological principles. It targeted the child’s social and physical home environment by intervening with parental weight-related knowledge, beliefs, and skills for managing child obesogenic behaviors; and addressed families’ needs for community resources supportive of a healthy lifestyle. The two-year CHW-delivered intervention was structured based on the 5As model (Agenda, Assess, Advise, Assist, Arrange follow up) and included two in person sessions and two telephone follow-ups per year with the parent, with a personalized letter and print materials sent after each contact. Parents also received quarterly newsletters, Facebook messages, and invitations to community events. The attention-control comparison condition used the same format and contact time as the intervention condition, but targeted positive parenting skills. Measurements occurred at baseline, and at 6-, 12-, 18- and 24-month follow-up. Assessments included anthropometrics, accelerometry, global positioning system (GPS), and self-report surveys. The primary outcome was child body mass index (BMI) z score. Secondary outcomes were parent BMI; and parent and child diet, physical activity, sedentariness, and utilization of community resources supportive of a healthy lifestyle.

**Discussion:**

A CHW-delivered parent-focused lifestyle intervention may provide a translatable model for targeting the high priority public health problem of childhood obesity among low-income diverse communities. If demonstrated effective, this intervention has potential for high impact.

**Trial registration:**

ClinicalTrials NCT03028233. Registered January 23,2017. The trial was retrospectively registered.

## Background

The prevalence of obesity in the United States continues to increase and is a major threat to public health [1] given its association with multiple chronic diseases and health conditions, and a shorter life expectancy [2–4]. Approximately one third of U.S. children and two thirds of adults in the US are overweight or obese [5, 6]. In Worcester, Massachusetts (study site), the prevalence of childhood overweight/obesity in Grades 1, 4, 7 and 10 is higher (41.3%) than the overweight/obesity prevalence in the state (31.3%) [7]. Socioeconomically disadvantaged and racial/ethnic minority groups are at a greater risk of being overweight or obese compared to non-Latino Whites [6, 8, 9]. Given that obese children are more likely to become obese adults [10], addressing obesogenic behaviors among children is key to long-term obesity prevention, particularly among at risk populations [8, 11, 12].

National organizations have addressed the importance of obesity prevention among children and have provided weight related behavior recommendations [13–15]. The American Academy of Pediatrics provides specific dietary and activity recommendations to help families in promoting a healthy weight and preventing obesity among their children [15–17]. These recommendations include reducing sugar sweetened beverages and high calorie snacks, as well as, reducing opportunities for sedentary screen time use and increasing physical activity [18–21]. However, strategies to disseminate these recommendations and for overcoming challenges to their implementation in disadvantaged and minority communities are needed.

The social ecological model posits that health behaviors are impacted by multiple levels of influence [22]. At the family/home level, the social and physical environments in the home have been identified as important targets to address child obesogenic behaviors [23–27]. Children’s eating and physical activities are influenced by their parents’ behaviors (including modeling) and parental decisions regarding food selection, preparation and availability in the home, among other [28–30]. Children from low-income families spend more time watching television and have parents that are less likely to model healthy eating behaviors compared to children from higher income families [31, 32]. At the community level, resources such as availability of markets, walkability of neighborhoods, transportation, parks and green space are associated with healthy diet and activity behaviors [33, 34]. Residents in diverse low-income neighborhoods may have fewer means to access resources outside of their neighborhood [35, 36], thus awareness and utilization of the existing community resources available to them may be critical to their adoption of healthier lifestyles. Few childhood obesity interventions to date targeted the home environment and the utilization of existing community resources [37–42].

Community health workers (CHW) are public health workers who are trusted members of a linguistic or cultural community with an in-depth understanding of the community they serve [43]. Frequently functioning as lay health advisors or patient navigators, they serve as a link between health care providers and community members; provide health education, social support and advocacy; and help increase health knowledge/literacy and self-efficacy. As such, some studies suggest that CHWs can facilitate the tailoring of interventions to cultural and contextual factors and literacy needs of individuals in low-income diverse communities [44–48]. CHW-delivered interventions have potential to be sustainable and scalable to other communities across the country [49].

This paper describes the design and methods of Healthy Kids & Families, a community-based quasi-experimental trial of a parent-focused CHW-delivered intervention for childhood obesity prevention, in accordance with the Transparent Reporting of Evaluations with Nonrandomized Designs (TREND) guidelines [50].

### Study objectives

The aim of this community-based study was to test the effectiveness of Healthy Kids & Families, a parent-focused CHW-delivered intervention to promote and assist a healthier lifestyle to prevent childhood obesity among low-income and minority families.

## Methods

### Study design

This quasi-experimental study was conducted in Worcester, Massachusetts through a partnership between the UMass Worcester Prevention Research Center, the Worcester Public Schools and Oak Hill Community Development Corporation. Nine elementary schools (Kindergarten to 6th grade) collaborated in the study, with schools being the unit of intervention allocation, paired based on demographic characteristics and location. Measures were collected from parent-child dyads at baseline, and at 6-, 12-, 18-, and 24-month follow up. Community partners contributed to the design of the study, including recruitment procedures and the selection of a topic for the attention-control comparison condition, and facilitated office space in the community for the conduct of the study assessments. The project was approved by the Institutional Review Board of the University of Massachusetts Medical School and the Worcester Public School Research Committee, with plans to communicate protocol changes to the study sponsor, IRB, participants and community partners.

### Study setting and population

The study was conducted in Worcester, Massachusetts, a city comprised of numerous low-income and racial/ethnically diverse neighborhoods. Parent-child dyads were recruited from 4 schools allocated to the intervention, and 5 schools allocated to the attention-control comparison condition between June 2015 and May 2017. Children were in kindergarten – sixth grade (over the age of 4). *Inclusion criteria* included: 1) child attends a participating school, 2) parent has access to a telephone, 3) English or Spanish-speaking, and 4) plan to live in the neighborhood for at least two years. *Exclusion criteria* included: a medical condition or advice from a doctor that precludes the child from walking or eating fruits and vegetables. For families with more than one eligible child attending the school, the child whose birthday was closest to the recruitment date was considered the index child and invited to join the study. The parent that communicated with the study by responding to the interest survey was the parent selected for participating in the study.

### Screening and recruitment

Recruitment consisted of several strategies. First, a letter from the school principal describing the study, and a response card (in English and Spanish), were placed in each child’s backpack by school staff. The response card asked questions about interest in activities related to the study and requested contact information from interested parents. To optimize response from parents, a $10 gift card was offered for returning the response card. Additionally, school principals implemented automated telephone messages alerting parents about the letter and response card; and the research staff conducted brief study presentations about the study at school events (i.e., parent nights, family events, Parent Teacher Organization meetings), and were available to talk with parents at school drop-off and pick-up times. Study staff also spoke with parents at local after-school programs attended by the children.

**Fig. 1:**
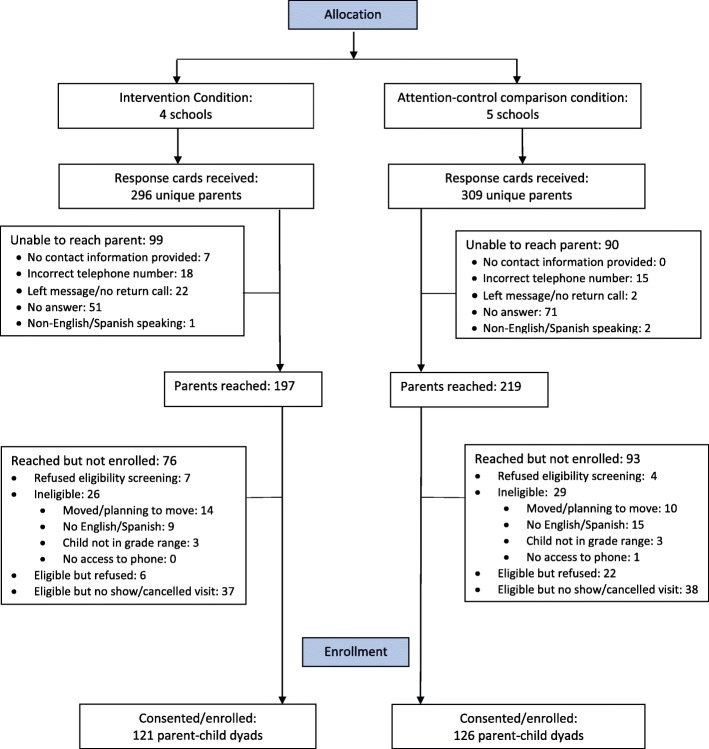
Recruitment and Enrollment

Our pool of potential participants (parents) was comprised of parents who returned the response card with their contact information. Upon receiving the response card from parents, a study recruiter attempted to contact each of these parents via the contact information provided on the response card to further explain the study, answer any questions, assess eligibility, and inquire about interest in study participation from eligible parents. Eligible and interested parents and their children were scheduled to attend a study visit. This visit was held at a community location, with transportation and adult supervision for children provided as needed. At this visit, the study was explained to the parent and the child and consent was obtained from the parent and assent was obtained from the child prior to collection of baseline measures. In all, 247 parent-child dyads were recruited to participate in the study. See Fig. 1 for the Recruitment and Enrollment diagram, Table 1 for Children’s Baseline Characteristics and Table 2 for Parent’s Baseline Characteristics.

**Table 1:** Children’s Baseline Characteristics

Total Number of Participants	N	247
Mean Age (SD)	247	7.8 (2.1)
Gender	247	
Male		127 (51.4%)
Female		120 (48.6%)
Race/Ethnicity	246	
White Non-Hispanic		37 (15.0%)
Black Non-Hispanic		41 (16.7%)
Asian Non-Hispanic		6 (2.4%)
More than one race/Other Non-Hispanic		11 (4.9%)
Hispanic		150 (61.0%)
Grade at Baseline	247	
Kindergarten		39 (16.6%)
Grade 1		43 (17.4%)
Grade 2		33 (13.4%)
Grade 3		44 (17.8%)
Grade 4		35 (14.2%)
Grade 5		26 (10.5%)
Grade 6		25 (10.1%)
BMI Z-Score (SD)	241	1.0 (1.2)
BMI Percentile (SD)	241	74.0 (27.9)

**Table 2:** Parent’s Baseline Characteristics

Total Number of Participants	N	247
Mean Age (SD)	247	36.2 (7.4)
Sex	247	
Male		21 (8.5%)
Female		226 (91.5%)
Race/Ethnicity	247	
White Non-Hispanic		55 (22.3%)
Black Non-Hispanic		42 (17.0%)
Asian Non-Hispanic		6 (2.4%)
More than one race/Other Non-Hispanic		13 (5.3%)
Hispanic		131 (53.0%)
Marital Status	245	
Single		86 (35.1%)
Married or living as married		116 (47.4%)
Separated/divorced/widowed		43 (17.5%)
Highest level of education	247	
Less than High School		47 (19.0%)
High School/GED		156 (63.2%)
More than High School		44 (17.8%)
Employment Status	245	
Employed		144 (58.8%)
Unemployed		37 (15.1%)
Disabled		19 (7.8%)
Homemaker		33 (13.5%)
Other		12 (4.9%)
Household Income	244	
<$20,000		135 (55.3%)
$20,000–$50,000		88 (36.1%)
>$50,000		21 (8.6%)
Receive Food Assistance	246	172 (69.9%)
Language primarily spoken at home	243	
English only		112 (46.1%)
More English than another language		29 (11.9%)
Both English and another language equally		31 (12.8%)
More another language than English		41 (16.9%)
Only another language		30 (12.4%)
Confidence filling out medical forms	246	
Extremely		131 (53.3%)
Quite a bit		66 (26.8%)
Somewhat		39 (15.9%)
A little bit/not confident/not at all confident		10 (4.1%)
BMI (SD)	240	31.9 (7.2)

### Intervention

#### Conceptual framework

The intervention was guided by social cognitive theory (SCT) [51] and social ecological principles [52–54]. As such, the intervention acknowledged that children’s behaviors are influenced by their family’s social and physical home environment, and that families are in turn influenced by contextual factors. Thus, the intervention targeted parent’s knowledge of behaviors that influence body weight; efficacy beliefs regarding management of child obesogenic behaviors; parental behavioral skills for change in diet, physical activity and sedentary behaviors; and parent’s knowledge of/needs for free community resources supportive of a healthy lifestyle (i.e., exercise classes, gyms, pools, healthy cooking classes, lunches, farmer’s markets, neighborhood and city parks, walking trails and others). Consistent with SCT, the intervention encouraged parents to set realistic goals for behavior change of the entire family involving modifications to the food and activity environment in the home; encouraged problem-solving of strategies to attain the set goals; and reminded parents that they can be positive role models for their children [55]. Additionally, neighborhood factors that could be challenges to a healthier lifestyle were acknowledged and families were made aware of, and encouraged to utilize, existing community resources supportive of their healthy lifestyle goals.

The family-centered intervention [56] delivery protocol was structured based on the 5As model, which includes setting a shared Agenda for all sessions; Assessing parent/family’s health-related values, beliefs and motivations for behavior change, history of prior change attempts and monitoring of progress; brief and personalized Advising regarding diet, physical activity and sedentary behaviors; Assisting parents with setting goals and developing an action plan for goal achievement including problem-solving anticipated challenges for change; and Arranging follow up [56–58]. This model was implemented using motivational interviewing principles that included open-ended questions, a non-judgmental attitude, understanding and working with ambivalence to change, and strategies to reduce resistance [59].

#### Behavioral targets

Intervention targets were chosen based on national recommendations for dietary and physical activity behaviors and research evidence [13–15, 18–21]. Diet targets included consumption of healthy low-calorie snacks, reduction of fast food, and reduction of sugar sweetened beverages. Activity targets included engagement in physical activity at least 60 min/day and reduction of screen time to less than 2 h/day. Messages related to these targets were summarized under the acronym “SUPER”: Snack Smart, Unplug and Play, Prepare and Plan, Energize with Exercise, and Rethink your Drink.

#### Intervention delivery

**Fig. 2:**
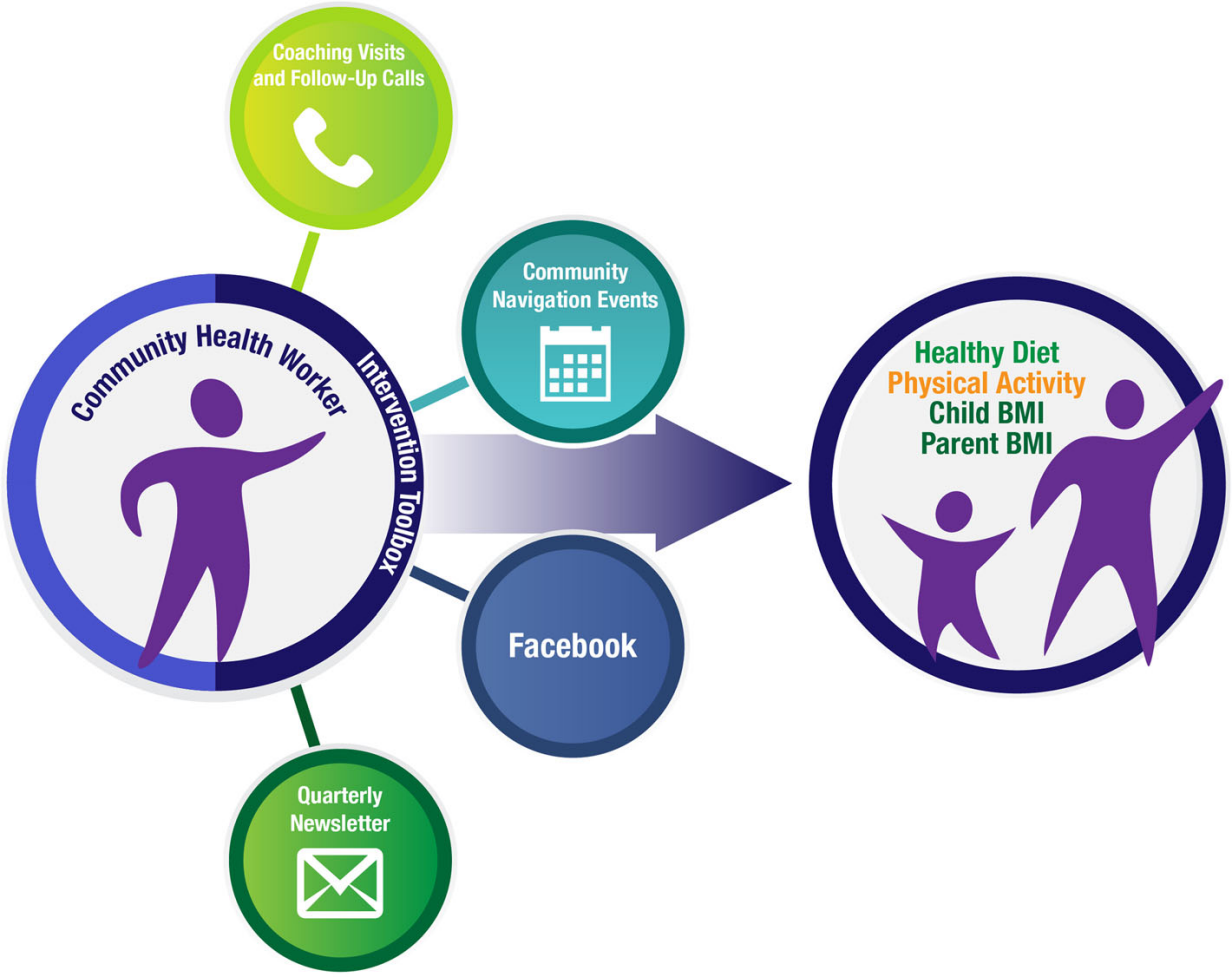
Healthy Kids & Families Model. This figure is our own image, but the images used to create it were taken from the stock photography website (istockphoto.com) with permission to use them. https://www.istockphoto.com/legal/license-agreement

The intervention was delivered by a trained CHW, serving as a coach, and is illustrated in Fig. 2. Briefly, over the course of two years, the CHW established and maintained contact with parents via two yearly one-hour in-person sessions offered at a location of their choosing (their home, study office, or other community location), and two yearly telephone follow-ups (alternating home and telephone contacts). A personalized letter was sent to the parent after each contact along with print materials related to the parent’s goals for the family. Parents also received monthly newsletters, Facebook messages, and mailed invitations to community events. All print materials were available in English and Spanish, and were culturally tailored and literacy-sensitive, and included pictures to illustrate main points.

**Fig. 3:**
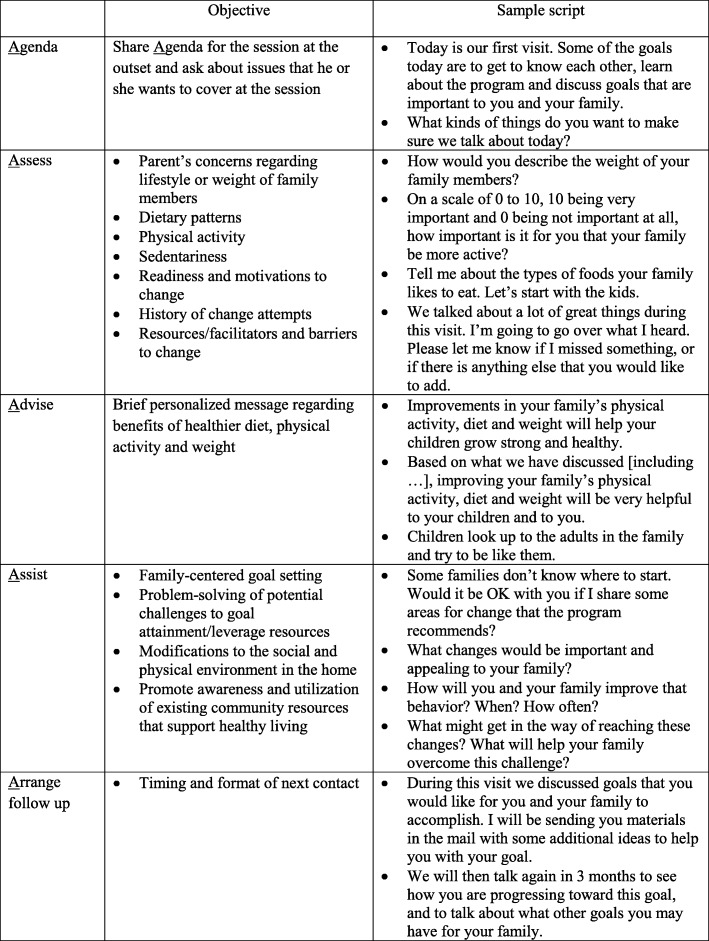
Adapted 5A protocol for the Lifestyle Intervention, Healthy Kids & Families Study

*In-person and telephone sessions:* Protocols for the in person and telephone-delivered sessions followed the 5As model (set Agenda, Assess, Advise, Assist, Arrange follow up) [60], and thus included a combination of structured and open ended questions implemented in accordance with principles of motivational interviewing (i.e., non-judgmental approach, elicit reflection on motivations to change and change talk) (Fig. 3). At the first session, parents received an intervention booklet which described the 5 main messages of the SUPER acronym and provided information about body mass index (BMI) for adults and children and goal setting. Follow-up calls assessed progress toward goals, facilitated problem-solving and new goal-setting, as appropriate, and reinforced intervention messages, and had an estimated duration of 20 min. After each in-person and telephone session, personalized mailings (English or Spanish) were sent to the parent. Mailings included a letter summarizing the family-centered goals set by the parent and print materials to support their goals, including culturally-tailored tip sheets and healthy recipes, and information on existing community resources (e.g., farmer’s market, YWCA, etc) related to the goals set.

*Community navigation events:* Community events supportive of a healthy lifestyle were organized throughout the year. Examples of events include grocery store tours for healthy shopping, and nature scavenger hunts at a local park. These events were led by the CHW every 3 months and were open to the entire family to attend.

*Newsletters:* Quarterly letters shared tips for behavior changes consistent with study messages, recipes for healthy meals, invitations to community events organized by the study, and information about free events sponsored by other community sources, such as calendar of free summer lunches for children, Farmer’s Markets schedules, and fitness opportunities.

*Facebook messages:* All interested participants were invited to join the study’s private (secret) Facebook page, accessible only via invitation. An initial 6 week Facebook campaign delivered the intervention messages through daily posts, with each week focusing on a specific targeted (SUPER) behavior. A trained CHW encouraged dialogue among participants on the Facebook page. Following the initial campaign, periodic posts shared information about community events as described above.

#### Intervention Fidelity

In order to maximize intervention fidelity, several measures were taken. The intervention protocol was manualized and included scripts for all intervention sessions and an appendix with print intervention materials and descriptions of existing community resources. The CHWs received training in the rationale for the study, principles of motivational interviewing, and implementation of the intervention following protocols in the intervention manual. Trainings involved a didactic component as well as practice in intervention delivery via role plays with corrective feedback from two members of the research team. Following training, intervention sessions were audio recorded and 10% of the recordings were randomly chosen for fidelity checks at weekly CHW supervision meetings using a session checklist. The CHW received feedback on errors of commission or omission, with further role plays conducted as needed.

### Attention-control comparison condition

The attention control comparison condition consisted of a CHW-delivered intervention focused on positive parenting. This condition followed the same format as the active lifestyle intervention. The topic of this control condition was decided upon through collaboration with school principals. The parenting program aimed to reinforce parental skills to facilitate child development of positive relationships, attitudes and behaviors. Summarized by the acronym “STARS”, key messages were: **S**et clear rules and be consistent, **T**each your children to solve conflicts, **A**ct to give a good example, **R**eward positive behavior, and **S**trengthen your support.

### Outcomes/measures

The primary outcome was child BMI z-score classified according to the CDC’s US children growth chart [61]. Secondary outcomes included parent BMI, calculated from body weight and height as weight (kg)/height(meters) [62]; and parent and child diet and sedentary behaviors assessed by self-reported (survey), and by ActiGraph GT 3.0. Utilization of community resources and mobility patterns were measured using global positioning system (GPS) devices. Assessments were completed at baseline, and at 6-, 12-, 18- and 24 follow-up. The 6 and 18-month assessments were brief (anthropometrics). Study assessors were rigorously trained in the administration of the study measures by members of the research team and assessment visits were audio-recorded and 10% of them reviewed for quality assurance and improvement purposes. Blinding was not possible given the nature of the study. To ensure comprehension by participants with varying literacy levels, survey measures were verbally administered in English or Spanish. Participants were compensated for their time. Upon completion of baseline, 12 and 24 month assessments, parents were given a $60 gift card, and children were given a small toy. Upon completion of the shorter 6 and 18 month assessments, parents were given a $20 gift card. Study measures are described in Table 3.

**Table 3:** Measurement Description

Variable	Measure	Sample item
Anthropometric and Health-related Measures		
BMI	From weight measured with digital Tanita BWB- 800 scale and height measured with SECA 213 stadiometer, with light clothing and no shoes. • Child BMI score is calculated as *BMIz*=[(BMI*M*)L−1] ÷ (L × S) [64] • Parent BMI calculated as weight (kg)/height(meters) [62].	
Waist circumference	Child and parent • Waist circumference measured twice (and averaged) with a non-stretchable measuring tape, following a standardized protocol [65].	
Blood pressure	• Blood pressure measured three times with a DinamapPro100, following a consistent protocol of sitting for 10 min before the first measure, and waiting at least 1 min in between each measure [66].	
Medications	Child and parent: Prescription and non-prescription medications and their dosage recorded by staff at study visits.	
Healthcare utilization and health conditions	• Child and parent health care utilization assessed by 12-item investigator developed survey. • General health perception assessed by 1-item from the RAND survey [67]. • Selected health conditions assessed by 28-item investigator-developed survey.	Does your child have a pediatrician, primary health care provider or a doctor? Do you have a history of high blood sugar or diabetes?
Behavioral Measures		
Physical activity and sedentariness	Child and parent • # Days > 60 mins physical activity assessed by 1 item from the MA: Parent Child Longitudinal Cohort Survey (MA CORD [68] (original from Youth Risk Behavioral Study [69]. • General moderate to vigorous physical activity levels during the school year assessed via a 7-day recall of activities modeled after items 1 and 9 of the Physical Activity Questionnaire (PAQ-C) [70, 71]. • Walking for transportation assessed by a 4-item investigator-developed survey (parent survey included additional question on walking to work). • Minutes and intensity of physical activity/day assessed via accelerometry (ActiGraph GT 3.0) [72, 73]. • Sedentary behavior assessed by 2-item investigator-developed survey.	During the past 7 days, on how many days was your child active for at least 60 min a day? During the past week, did you walk for exercise? If yes, how much time did you walk for exercise on: Monday-Friday, hours/minutes
Diet	Child and parent • Vegetables, fruit, and fast food consumption assessed by 3-items adapted from the MA CORD study [68]. • Snacks eaten at home assessed by the 10-item Beverage and Snack Questionnaire II [74]. • Beverage consumption assessed by 25 items modified from the Beverage Intake Questionnaire [75]. The survey measure asked about milk, soda and other beverages and parents were asked about alcoholic beverages.	How often did you usually eat [name of food] in the past month? How many times did you eat regular chips when you were at home this past week? How often did you drink whole milk in the past month? How much did you typically drink each time?
Tobacco	Parent • Tobacco use assessed by 2-items from the Behavioral Risk Factor Surveillance System [76] and 2-items from the National Health and Nutrition Examination Survey Questionnaire [77]	Have you smoked at least 100 cigarettes in your entire life?
Mobility patterns	Child and parent • Utilization of community resources and mobility patterns were assessed by a GPS unit [78]	
Demographic and Other Characteristics		
	Child and parent • Demographics assessed by 11-items from the MA CORD study about: race, ethnicity, nativity, marital status (parent), pregnancy status (parent), language, and income [68]. • Gender assessed by 1-item on parent and child gender from We Heart Health Literacy [79]. • Language, years in the US, education, employment, health insurance, housing and living arrangements, missed work/school, child grade and school, use of technology, assessed by investigator-developed items. • Literacy assessed by 1-item from Chew et al. [80], and Wallace et al. [81]. • Food insecurity assessed by 1-item from the MA CORD study (parent) [68] (originally from Behavior Risk Factor Surveillance System) [82]. • Child education-related needs assessed by 2-item investigator-developed survey • Perceptions (parent) of neighborhood characteristics that could interfere with walking and biking assessed by 4-item investigator-developed survey • Access to fruits and vegetables assessed by 2-items from Neighborhood Scales [83]	What languages do you speak? Did you attend elementary or grade school at all, either here or in another country? How confident are you filling out medical forms by yourself?
Parent Psychosocial Measures		
Weight management literacy	• Knowledge of weight management assessed by 29-item scale developed by investigators.	Drinking water instead of juice can help a person lose weight.
Weight-related attitudes and perceptions	• Body image assessed by 4-item survey developed by Collins [84] • Weight satisfaction assessed by 5-item investigator-developed survey • Parent description of child weight assessed by 1-item survey adapted from MA CORD Study [68]. • Readiness for weight loss effort (parent) assessed by 12-item investigator-developed survey [85].	Please point to the picture that shows the way you want to look. How satisfied are you with your weight? In the past six months, have you made a serious attempt to avoid gaining weight? How would you describe your child’s weight? Would you say that he or she is...
Social norms for lifestyle behaviors and weight	• Social norms for diet, physical activity, screen use and bed time assessed by 10-item survey adapted from the MA CORD study [68]. • Social norms for healthy weight assessed by investigator-developed item.	How many of the people you know give children snacks any time they ask?
Self-efficacy	• Self-efficacy for positive parenting assessed by 8-item investigator-developed survey.	How confident are you in your ability to teach your child to solve conflicts?
Engagement	• Engagement assessed by 1-item from the Harvard Family Research Project [86] • Engagement with other parents assessed by 1-item investigator-initiated survey	How often do you meet in person with teachers at your child’s school?
Effective parenting skills	• Effective parenting assessed by 9-items adapted from the 42-item Alabama Parenting Questionnaire [87].	How often do you let [child’s name] know when he/she is doing a good job with something?
Depression Symptoms	• Depression symptoms assessed by Center for Epidemiologic Studies Depression Scale (CESD) [88].	How often have you felt depressed during the past week.
Parent-reported Child Behaviors and Emotions		
Child behavior	• Child behavior assessed by 6-items modeled after selected content adapted from the 36-item Eyberg Child Behavior Inventory [89].	How often does [child’s name] throw a fit, or get very angry?
Emotional regulation	• Emotional regulation assessed by 2-item investigator-developed survey.	How often does [child’s name] manage his or her emotions in a way that is appropriate for his or her age?
Child body image	• Body image assessed by 2-item survey developed by Collins [84]	Point to the picture that shows the way you think is best for a child your age to look.

All measures were adapted for verbal administration in English and Spanish.

### Process measures

The study included process data, such as recruitment and assessment contact attempts (number/mode i.e., phone, text, email, Facebook), and completion and retention rates. The study also included intervention process data, such as contact attempts (number/mode), session completion rate, goals set and attained, community resource referrals, and program satisfaction.

### Participant retention

Multiple methods were used to maximize participant retention. Upon enrollment, participants were asked to provide complete contact information including telephone numbers, email addresses, and Facebook name, with information updated at each subsequent contact. Participants also provided contact information for a friend or family member who could reach them if the study staff were unable to establish contact with them. An electronic tracking system identified participants due for intervention sessions and assessments, prompting staff to contact participants during the intervention or assessment window. Staff were able to record a summary of each contact with each participant, including situations shared by participants that could be followed up at the next contact (i.e., a sick family member, difficulties at work, an upcoming vacation, etc.) to support continuity. Staff were trained in motivational interviewing skills, in effort to maximize rapport and identify and support participant motivations to participate. Participants were offered options for completing the intervention sessions and assessments visits at a convenient time (including evenings and weekends) and location (one of two study offices, or at their home), and holiday and birthday cards were mailed to all participants. Lastly, at weekly intervention and assessment staff meetings, participants that were “hard to reach” were identified and the staff brainstormed, on a case by case basis, ways to maintain engagement with participants.

### Data management and quality assurance

Study databases used the secure, web-based REDCap (Research Electronic Data Capture) [63] hosted at UMass Medical School, and included a participant tracking system, an intervention tracking system and assessment data storage. The study also used QuickBase, a web-based software that utilized algorithms to prompt the study staff when participants were due for an assessment or an intervention visit. Weekly intervention and assessment contact and completion data were downloaded from REDCap and uploaded to QuickBase.

For quality assurance, intervention and assessment sessions were audio recorded with participant permission as described above. Procedures to monitor and respond to elevated blood pressure readings and self-report of suicidal ideation or intent were in place and the study staff were trained in their implementation.

### Sample size calculations

Sample size calculations were based on a study design that included one baseline measure and 4 follow-up measures, the ability to detect an intervention effect of 0.098 BMI z-score using ANCOVA (or linear mixed models) method at 5% significance level and at least 80% power. Based on literature review, the mean (SD) of BMI z-scores for the intervention and control groups were assumed to be 0.97 (1.0) and 0.86 (1.0), respectively, and a serial correlation of 0.95. With these assumptions, the study aimed to enroll a sample size of 240 participants and expected complete data on 200 participants (17% loss to follow up) at study completion. The sample size was estimated using Stata MP 12 (Stata Corp., College Station, TX).

### Dissemination

The dissemination plan included presentations and availability of intervention protocols and materials to local and regional community-based organizations including the Worcester Public Schools, and dissemination to the academic community through publications in academic journals and presentations in professional conferences. The plan also included sharing results with study participants at a specially organized event upon study completion.

### Analysis

The analytic plan included generalized linear mixed models to determine the effectiveness of the intervention compared to the attention control comparison condition, on child and parent BMI and physical activity and diet outcomes. To account for auto-correlations among repeated measures on the same participant, the plan included in the mixed models either first order autoregressive correlation or unstructured covariance structure for robust estimates. Estimates for the intervention effects were made with and without adjusting for child, family and neighborhood covariates. To test whether intervention effects varied by these covariates, the plan included tests of the significance of the interactions between intervention indicators and each covariate. A significant interaction term would signal potential variation of intervention effect, warranting further investigation. To understand parental influences on child behavior and obesity, the plan included combining child and parent data to analyze longitudinal changes in child behaviors and BMI in relation to their parents’ changes in perceptions, eating and activity behaviors and BMI. An intention to treat analysis for all participants enrolled in the study was planned according to their treatment assignment. To account for missing data, multiple imputation methods were planned, as were inverse probability weighting methods to account for participant dropouts. Planned analyses also included beginning to assess the sustainability of the intervention.

## Discussion

This paper described the design and methods of a quasi-experimental trial of Healthy Kids & Families, a parent-focused, CHW-delivered intervention to assist low-income and minority families adopt a healthier lifestyle to prevent childhood obesity. The intervention promoted changes in child diet and activity behaviors in accordance with research evidence and recommendations from national organizations for preventing childhood obesity [13–15, 18–21]. Should study findings warrant it, the CHW-intervention model has potential for dissemination and sustainability.

The study has limitations and potential weaknesses. Given the nature of the study, it was not possible to blind study participants to intervention condition. Similarly, although study assessors were not informed about participants’ study condition, it is possible that participants could have shared this information with the assessors during the assessment visits. There were at least two potential sources of contamination. The first one being that the same CHW delivered the intervention and the control condition. To reduce this risk the CHW was rigorously trained to understand the risk of contamination and strategies to minimize this risk. The CHW was closely supervised by study staff and session notes and goals set during sessions were reviewed for all participants at all sessions. Additionally, calls were recorded with parent permission. The second potential source of contamination involved the potential for families in the intervention condition sharing information about their intervention with families from the other condition. This potential for contamination was minimized by working with neighborhood schools with geographic boundaries (the study did not include magnet schools, which serve children from all parts of the city). This reduced the risk that families knew each other through neighborhood activities and programs. Other limitations include the potential for self-selection bias. For example, given that participation was voluntary, parents with concerns about their children’s weight or their behavior (focus of the intervention and control conditions, respectively) may have been more interested in the study. To reduce this bias, our recruitment effort provided a small monetary incentive to all parents in the schools for completing a brief interest survey, whether or not they were interested in participating in the study. Our recruitment pool was comprised of parents who responded to the survey.

The focus of the study was limited to examining the effectiveness the intervention and it did not assess cost or cost effectiveness of the intervention. Potential risk of harm from the intervention was possible as families may have had unrealistic expectations for improving their health and these expectations may have gone unfulfilled. We attempted to mitigate this risk through efforts to facilitate appropriate understanding of the study conditions prior to and during the consent process and providing ample opportunities for participants to ask questions prior to study enrollment. Additionally, both intervention and control conditions followed the 5A algorithm and thus an important component of the intervention involved helping participants set realistic goals for themselves and their families and brainstorming facilitators and challenges to goal attainment to plan appropriate solutions. Finally, there were differences in BMI z-scores among the schools thus the analytic plan will account for baseline site difference by inclusion of an intercept for each site. Due to lack of pilot data, this was not considered in the original sample size calculation.

## Abbreviations

5As: Agenda, Assess, Advise, Assist, Arrange
BMI: Body mass index
CHW: Community health worker
GPS: Global positioning system
SCT: Social cognitive theory
